# Regulatory T cell enhancement in adults with cystic fibrosis receiving Elexacaftor/Tezacaftor/Ivacaftor therapy

**DOI:** 10.3389/fimmu.2023.1107437

**Published:** 2023-02-16

**Authors:** Dirk Westhölter, Jonas Raspe, Hendrik Uebner, Johannes Pipping, Mona Schmitz, Svenja Straßburg, Sivagurunathan Sutharsan, Matthias Welsner, Christian Taube, Sebastian Reuter

**Affiliations:** ^1^ Department of Pulmonary Medicine, University Hospital Essen- Ruhrlandklinik, Essen, Germany; ^2^ Adult Cystic Fibrosis Center, Department of Pulmonary Medicine, University Hospital Essen- Ruhrlandklinik, Essen, Germany

**Keywords:** pulmonary infection, cytokines, immunophenotyping, CFTR modulator therapy, cystic fibrosis - immunology, Pseudomonas aeruginosa

## Abstract

**Introduction:**

Cystic fibrosis (CF), especially CF lung disease, is characterized by chronic infection, immune dysfunction including impairment of regulatory T cells (Tregs) and an exaggerated inflammatory response. CF transmembrane conductance regulator (CFTR) modulators have shown to improve clinical outcomes in people with CF (PwCF) with a wide range of CFTR mutations. However, it remains unclear whether CFTR modulator therapy also affects CF-associated inflammation. We aimed to examine the effect of elexacaftor/tezacaftor/ivacaftor therapy on lymphocyte subsets and systemic cytokines in PwCF.

**Methods:**

Peripheral blood mononuclear cells and plasma were collected before and at three and six months after the initiation of elexacaftor/tezacaftor/ivacaftor therapy; lymphocyte subsets and systemic cytokines were determined using flow cytometry.

**Results:**

Elexacaftor/tezacaftor/ivacaftor treatment was initiated in 77 PwCF and improved percent predicted FEV1 by 12.5 points (p<0.001) at 3 months. During elexacaftor/tezacaftor/ivacaftor therapy, percentages of Tregs were enhanced (+18.7%, p<0.001), with an increased proportion of Tregs expressing CD39 as a marker of stability (+14.4%, p<0.001). Treg enhancement was more pronounced in PwCF clearing Pseudomonas aeruginosa infection. Only minor, non-significant shifts were observed among Th1-, Th2- and Th17-expressing effector T helper cells. These results were stable at 3- and 6-month follow-up. Cytokine measurements showed a significant decrease in interleukin-6 levels during treatment with elexacaftor/tezacaftor/ivacaftor (–50.2%, p<0.001).

**Conclusion:**

Treatment with elexacaftor/tezacaftor/ivacaftor was associated with an increased percentage of Tregs, especially in PwCF clearing Pseudomonas aeruginosa infection. Targeting Treg homeostasis is a therapeutic option for PwCF with persistent Treg impairment.

## 1 Introduction

Cystic fibrosis (CF) is caused by mutations in the CF transmembrane conductance regulator (CFTR) gene resulting in dysfunctional or absent CFTR protein. CFTR modulators directly target this underlying protein defect and improve clinical outcomes in individuals with a wide range of CFTR mutations. Elexacaftor/tezacaftor/ivacaftor (ELX/TEZ/IVA) is the first approved triple combination CFTR modulator therapy that is indicated for people with CF (PwCF) who have a F508del mutation in at least one allele (1, 2).

While recent studies analyzing the efficacy of CFTR modulators have focused on clinical outcome parameters, there is a relative lack of data about whether CFTR modulators also dampen CF-associated inflammation. Exaggerated inflammation is a major driver of disease progression in CF that primarily, but not exclusively, affects the lungs (3). Increased levels of acute-phase proteins and pro-inflammatory cytokines are also present in peripheral blood from PwCF (3–5). Among these, interleukin [IL]-6, IL-8 and IL-1β have been reported to be elevated in the systemic circulation of PwCF compared with healthy controls (4, 6, 7). In addition, T cells are involved in inducing and maintaining chronic inflammation in CF (8, 9). Regulatory T cells (Tregs), which are known to counterbalance inflammatory processes and help to maintain homeostasis between pro- and anti-inflammatory mediators, are reduced in peripheral blood from PWCF (10, 11). CFTR dysfunction and chronic *Pseudomonas aeruginosa* infection are major mediators of the reported Treg impairment (10).

Given that CFTR is widely expressed on epithelial cells and on immune cells, highly effective CFTR modulators might resolve immune dysregulation and inflammation that result from a lack of CFTR activity. Current evidence on the potential anti-inflammatory effects of CFTR modulators is limited and is predominantly based on studies investigating the effects of mono- (ivacaftor) or dual combination (ivacaftor/lumacaftor, ivacaftor/tezacaftor) CFTR modulators (12, 13). Decreased sputum levels of IL-8 and IL-1β, and decreased serum levels of tumor necrosis factor (TNF) and IL-1β have been reported in PwCF receiving ivacaftor or ivacaftor/tezacaftor, respectively (14–16). However, these studies involve only a small number of participants and conflicting data exist (17). We previously compared PwCF receiving versus not receiving mono or dual combination CFTR modulators in a cross-sectional study and did not find any difference in either lymphocyte subsets including Tregs or cytokines in peripheral blood (18).

Available lines of evidence indicate a significantly higher efficacy of the triple combination compared with dual combination therapies with ivacaftor/teazacaftor or ivacaftor/lumacaftor in PwCF homozygous for the F508del mutation (2, 19). We therefore hypothesized that there would be effects on the composition of immune cell subsets and systemic cytokines in PwCF receiving highly effective triple combination CFTR modulators. Therefore, this longitudinal, observational study analyzed the effects of treatment with ELX/TEZ/IVA on immune cell subsets and systemic cytokines in PwCF

## 2 Methods

### 2.1 Study design and participants

Eligible participants were all PwCF starting on ELX/TEZ/IVA (T0) between March 2020 and January 2021. Those with clinical signs of a pulmonary exacerbation were excluded. Study participants were invited to follow-up consultations including pulmonary function tests, six-minute walk test and sweat chloride measurement at three (T1) and six (T2) months after treatment initiation. Results from sputum and throat swab cultures provided during 12 months treatment with ELX/TEZ/IVA therapy were studied retrospectively. *P. aeruginosa* infection status was classified according to Leeds criteria: chronic *P. aeruginosa* infection was defined as >50% of months with *P. aeruginosa*-positive sputum cultures in the preceding 12 months. Written informed consent was obtained from all study participants prior to enrolment. This study was approved by the local ethics committee (no. 17-7365-BO).

### 2.2 PBMC isolation

Whole blood samples were collected at T0, T1 and T2 and prepared as previously described (18). In brief, 12 mL of whole blood was collected from each participant and processed within four hours after collection. Following centrifugation, plasma samples (for cytokine measurement) and peripheral blood mononuclear cells (PBMC, for lymphocyte phenotyping) were obtained. PBMC were then adjusted to 1x10^7^ cells/mL phosphate-buffered saline (PBS) for further use.

### 2.3 Lymphocyte phenotyping

PBMC were adjusted to 2x10^5^ cells and washed with flow cytometry wash buffer (4% PFA [Carl Roth, Karlsruhe, Germany], 1% fetal calf serum [FCS; PAN-Biotech, Aidenbach, Germany] in PBS). PBMC were then incubated with a mix of antibodies purchased from BioLegend^®^ (Koblenz, Germany) for multicolor cytofluorometric analyses. Antibodies and the gating strategy used to identify T cell subsets have been described previously and were adapted from Rühle et al. (**Figure S1**; 18, 20). Tregs were defined as CD4^+^CD127^low^CD25^+^ cells and further characterized as CD39^+^ “stable” Tregs (**Figure S1**). In addition, Tregs were subdivided into FoxP3^+^ (BioLegend^®^ PE α-human FoxP3 clone:206D) Tregs in stored samples from 10 PwCF (before and 3 months after receiving ELX/TEZ/IVA, **Figure S2**). Intracellular staining against the transcription factors FoxP3 was performed on surface-stained PBMC using the FoxP3/Transcription Factor Staining Buffer Set (Invitrogen^®^, Darmstadt, Germany). Flow cytometric measurements were performed with a CytoFLEX LX (Beckman Coulter, Krefeld, Germany) and corresponding software (CytExpert V2.3). Finally, data were analyzed using FlowJo Software V10 (Tree Star, Ashland, USA).

### 2.4 Quantification of cytokines

Concentrations of T helper cell-associated cytokines were quantified in plasma samples from PwCF before and after 3 months’ treatment with ELX/TEZ/IVA using the bead-based immunoassay LEGENDplex^®^ (LEGENDplex^®^ Human Th Panel Standard V02, BioLegend, Koblenz, Germany) according to the manufacturers’ instructions (21). Samples were processed as described previously (18).

### 2.5 Statistical analyses

Two-tailed (un)paired Student-t-test was used for parametric data. Nonparametric data were analysed using the Mann–Whitney U-test for unpaired data or the Wilcoxon signed-rank test for paired data. Pearson Chi-squared test was used to assess frequency distributions of categorical data. Correlations were analysed using the pairwise Spearman correlation test. Data are displayed as mean and standard deviation or median with first and third quartile, as indicated. Statistical significance was defined as p<0.05. GraphPadPrism version 9, IBM SPSS version 28 and/or R studio (version 1.4.1106) were used for statistical operations. Plots and graphs were generated with various R packages (“ggplot2”, “ggiraph”, “ggpubr”, “factoextra”, “ggradar”, “fmsb”) in its latest versions as of August 2022.

## 3 Results

### 3.1 Study population

The study population consisted of 77 PwCF (mean age 34 years). The majority of PwCF (74/77, 96.10%) were either homozygous or heterozygous for the F508del mutation. Three PwCF with other CFTR mutations received off-label treatment with ELX/TEZ/IVA. Although ELX/TEZ/IVA therapy was not initiated in PwCF with recent pulmonary exacerbation, several PwCF showed signs of systemic inflammation at T0, as shown by elevated C-reactive protein (mean 1.25 mg/dl) and leukocytes (mean 10.60/nL) (**Table 1**). Among analyzed PwCF 59/77 (76.62%) received either inhaled antibiotics, intravenous antibiotic treatment in the last 12 months or both.

**Table 1:** Study population and results from immunophenotyping measurements.

	0 (n=77)	T1 (n=77)	T2 (n=77)	p-value (T0-T1)	p-value (T1-T2)	p-value (T0-T2)
Age, years	33.77 ± 11.52					
Female sex, n (%)	34 (44.2)					
ppFEV_1_	44.86 ± 20.27	57.37 ± 22.33	56.92 ± 22.11	**<0.001**	0.261	**<0.001**
BMI, kg/m^2^	20.09 ± 2.58	21.13 ± 2.69	21.64 ± 2.63	**<0.001**	**<0.001**	**<0.001**
Six-minute walk test, m	493.4 ± 104.7	578.1 ± 118.6	589.2 ± 114.7	**<0.001**	0.079	**<0.001**
Sweat chloride, mmol/L	105.52 ± 12.16	56.37 ± 19.06	52.24 ± 17.97	**<0.001**	0.083	**<0.001**
Leukocytes,/nL	10.60 ± 3.50	7.34 ± 2.49	7.75 ± 2.63	**<0.001**	**0.046**	**<0.001**
CRP, mg/dL	1.25 ± 1.81	0.02 ± 0.13	0.05 ± 0.25	**<0.001**	0.864	**<0.001**
*P. aeruginosa* infection, n (%)						
Chronic	37 (48.1)					
Non chronic	40 (52.0)					
CFTR genotype, n (%)						
Homozygous dF508	40 (52.0)					
Heterozygous dF508	34 (44.2)					
Other*	3 (3.9)					
Prior CFTR modulator therapy, n (%)						
Tezacaftor/Ivacaftor	34 (44.2)					
Lumacaftor/Ivacaftor	3 (3.9)					
Ivacaftor	0 (0.0)					
None	40 (52.0)					
Immunophenotyping						
CD3^+^ T cells, % PBMC	42.20 ± 16.65	42.58 ± 15.35	45.23 ± 13.81	0.831	0.126	0.128
CD8^+^ T cells, % T cells	24.50 ± 8.52	26.10 ± 7.67	25.48 ± 6.90	**0.037**	0.240	0.168
CD4^+^ T helper, % T cells	63.65 ± 10.40	62.29 ± 10.35	63.16 ± 8.99	0.121	0.279	0.602
CD25^+^CD127^-^ Treg, % T_h_	8.30 ± 2.37	9.85 ± 3.18	9.70 ± 2.75	**<0.001**	0.645	**<0.001**
CD39^+^ Treg, % Treg	45.16 ± 14.06	51.68 ± 12.72	51.84 ± 11.41	**<0.001**	0.895	**<0.001**
FoxP3^+^ Treg, % Treg**	82.39 ± 6.99	79.86 ± 10.38		0.221		
Effector T_h_, % T_h_	87.90 ± 3.11	86.69 ± 4.20	86.56 ± 3.70	**0.011**	0.781	**0.001**
T_h_1, % effector T_h_	10.02 ± 4.90	10.23 ± 4.39	11.15 ± 4.97	0.735	0.133	0.063
T_h_2, % effector T_h_	68.07 ± 13.14	67.60 ± 13.61	65.54 ± 14.21	0.648	0.106	0.221
T_h_17, % effector T_h_	10.14 ± 4.22	10.12 ± 4.39	9.47 ± 4.15	0.975	0.147	0.150
T_h_1-17, % effector T_h_	7.72 ± 4.57	7.80 ± 4.41	8.91 ± 5.47	0.792	0.071	0.087

Values are mean ± standard deviation. p-values were obtained using Wilcoxon test or t test for paired samples. BMI, body mass index; CFTR, cystic fibrosis transmembrane conductance regulator; CRP, C-reactive protein; PBMC, peripheral blood mononuclear cell; ppFEV_1_, percent predicted forced expiratory volume in 1 second; T_h_, helper T cells; Treg, regulatory T cells. *Other: R553X/I336K, G542X/3849+10KbC->T, R1162X/A455E. **FoxP3 expression determined in stored samples from a subcohort of 10 PwCF.

Characteristics at baseline (T0), at 3 months (T1) and at 6 months (T2).

Bold values denote statistical significance.

### 3.2 Clinical efficacy

After 3 months’ treatment with ELX/TEZ/IVA (T1), PwCF showed increased percent predicted FEV_1_ (ppFEV_1_; +12.5 points, p<0.001 vs. baseline), then ppFEV_1_ remained stable between T1 and T2 (p=0.261). Sweat chloride levels decreased by 46.6% between T0 and T1 (p<0.001), with sweat chloride levels below 30 mmol/L in seven PwCF (7/77, 9.1%) at T2 (**Table 1**). Furthermore, the three PwCF with off-label ELX/TEZ/IVA therapy showed improved ppFEV_1_ (+4.7 points) and reduced sweat chloride levels (-52.6%). Thirty-seven PwCF (37/77, 48.1%) had chronic pulmonary *P. aeruginosa* infection. At follow-up, sputum cultures were negative for *P. aeruginosa* in six of these participants (6/37, 16.2%) (**Table 2**).

**Table 2:** Sputum culture results.

Follow up results	Sputum culture (n=26)	Throat culture (n=10)	All (n=36)
Patients with >50% of cultures positive for *P. aeruginosa* (mean Treg increase T0-T1)= chronic	18 (+16.7%)	3 (+11.9%)	21 (+16.0%)
Patients with <50% of cultures positive for *P. aeruginosa* (mean Treg increase T0-T1)= intermittent	6 (+33.7%)	3 (+28.9%)	9 (+32.1%)
All negative, *P. aeruginosa* potentially cleared (mean Treg increase T0-T1)	2 (+43.8%)	4 (+37.8%)	6 (+39.8%)
p-value			**0.039**

Follow-up results from sputum and throat swab cultures and regulatory T cell (Treg) dynamics in PwCF with pre-treatment chronic P. aeruginosa infection status (n=37). At least two follow-up sputum samples or throat swabs were available from 36/37 (97.30%) study participants. PwCF who cleared P. aeruginosa infection were those with strongest Treg enhancement (+39.8%). Statistics: ANOVA.

Bold values denote statistical significance.

### 3.3 Effects of ELX/TEZ/IVA on immune cell subsets

**Figure 1:**
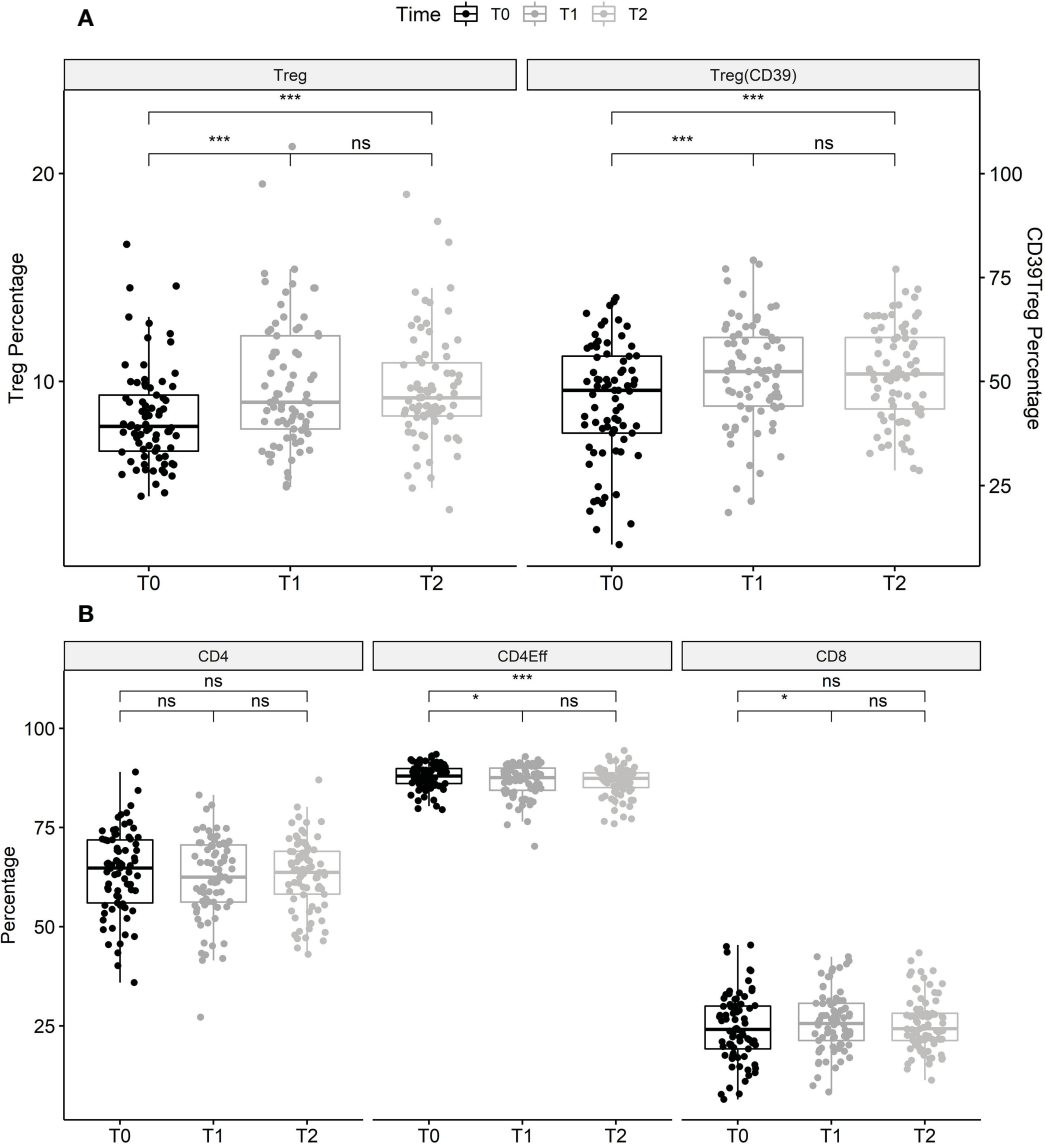
Changes of lymphocyte subsets during treatment with elexacaftor/tezacaftor/ivacaftor. **(A)** Data shown are the proportion of Tregs (CD4^+^, CD25^+^, CD127^-^ as a proportion of T helper cells) at T0, T1 and T2, and Tregs with CD39^+^ expression (as a proportion of total Tregs) as a marker of stability in pro-inflammatory environments. Tregs (+18.7%) and CD39^+^ Tregs (+14.4%) significantly increased between T0 and T1, and between T0 and T2, then remained stable between T1 and T2. **(B)**: CD4^+^ T helper (as a percentage of T cells), effector helper T cells (T_h;_ as a percentage of total T_h_) and CD8^+^ T cells (as a percentage of T cells) before, and after 3 months (T1) and 6 months (T2) of treatment with elexacaftor/tezacaftor/ivacaftor in PwCF. Percentages of effector T_h_ cells significantly decreased after initiation of elexacaftor/tezacaftor/ivacaftor therapy and remained lower at T2. Percentages of CD4^+^ and CD8^+^ T cells were stable between T0 and T2. Statistics: Values are median, Wilcoxon signed rank test. ns, not statistically significant. *p < 0.05; ***p < 0.001.

**Figure 2:**
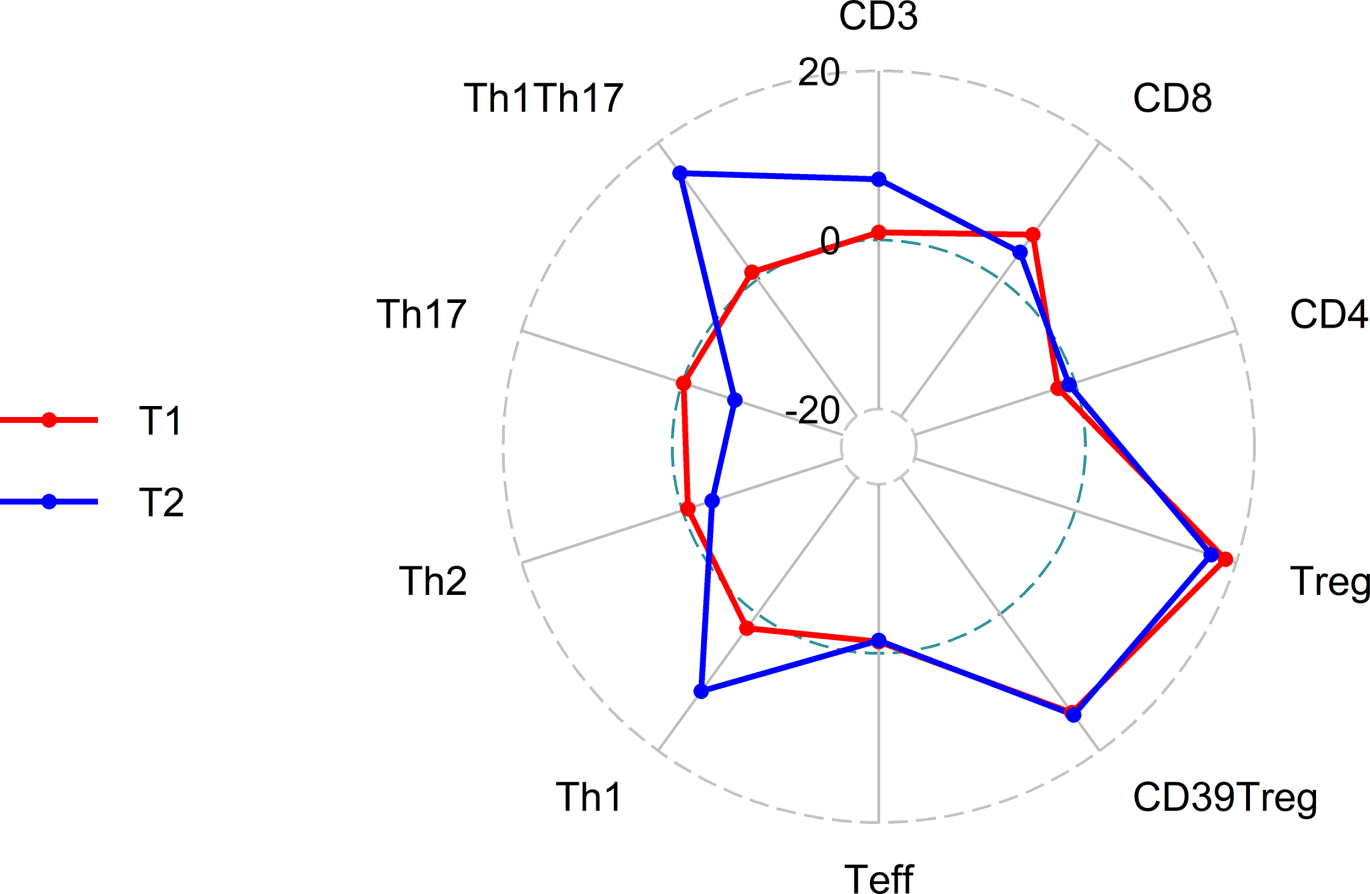
Radar plot of percentage change in lymphocyte subsets at T1 (3 months after initiation of elexacaftor/tezacaftor/ivacaftor, red) and T2 (6 months after initiation of elexacaftor/tezacaftor/ivacaftor, blue) compared with baseline (T0).

Treatment with ELX/TEZ/IVA had no significant effect on the proportion of total CD3^+^ T cells (mean 42.2% of PBMC at T0 vs. 42.6% at T1 and 45.2% at T2) or the proportion of CD4^+^ T helper cells (mean 63.7% of T cells at T0 vs. 62.3% at T1 and 63.2% at T2). Modest changes were observed in the percentage of CD8^+^ T cells between T0 and T1 (mean 24.5% vs. 26.1% of T cells; +6.5%, p=0.037), but not between T0 and T2 (p=0.168). However, treatment with ELX/TEZ/IVA was associated with a significant increase of Tregs (CD4^+^CD127^low^CD25^+^cells) from T0 to T1 (mean 8.3% vs. 9.9% of T helper cells, +18.7%; p<0.001), while percentages of Tregs remained stable between T1 and T2 (p=0.645; **Figure 1**). Treg enhancement was found in PwCF with elevated baseline CRP and leukocyte levels and in those with normal baseline CRP and leukocytes (**Figure S3**). CD39 further characterizes a stable subset of Tregs under inflammatory conditions (22). The proportion of CD39^+^Tregs disproportionately increased during ELX/TEZ/IVA therapy (mean 45.2% of Tregs at T0 vs. 51.7% at T1, +14.4%; p<0.001), with no significant change between T1 and T2 (p=0.895). FoxP3 expression of Tregs was determined in a subcohort of 10 PwCF. Overall, FoxP3 expression was found in 82.39% (mean) of Tregs at baseline and remained stable within the increased share of Tregs at T1(p=0.221). FoxP3 expression was higher in CD39^+^Tregs compared with CD39^-^Tregs at baseline (p=0.002) and T1 (p=0.007, **Figure S2**). ELX/TEZ/IVA therapy neither changed the FoxP3 expression among CD39^+^Tregs (p=0.846) nor among CD39^-^Tregs (p=0.770, **Figure S2**). Moreover, percentages of effector T helper cells decreased from T0 to T1 (mean 87.9% of T helper cells vs. 86.7%, –1.38%; p=0.011) and no changes were observed between T1 and T2 (**Figure 1**). Minor shifts occurred among effector T helper cell subpopulations. Percentages of Th1 effector cells tended to increase while effector T helper cells with Th17 phenotype tended to decrease. Th2 effector cells seem to be almost unaffected by treatment with ELX/TEZ/IVA (**Table 1** and **Figure 2**).

### 3.4 Effects of ELX/TEZ/IVA on *P. aeruginosa* infection status

**Figure 3:**
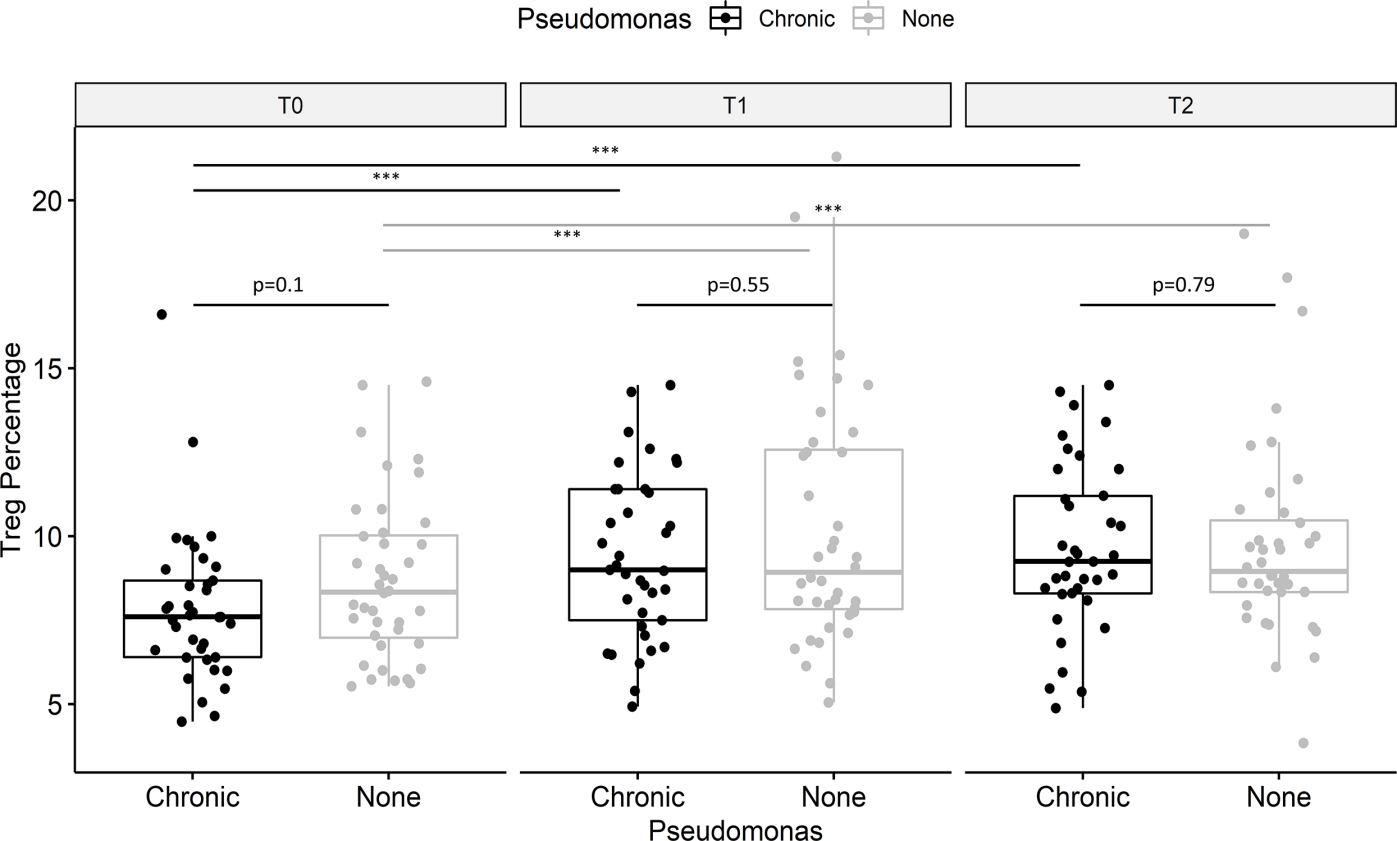
Effect of *Pseudomonas aeruginosa* infection on regulatory T cells (Tregs). Data showed are for CD25^+^ CD127^-^ Treg (as a proportion of T helper cells), stratified for *P. aeruginosa* infection. PwCF with chronic *P. aeruginosa* infection tended to have lower percentages of Tregs (black) compared to PwCF without chronic *P. aeruginosa* infection (grey) before therapy (T0). Treg impairment in PwCF with *P. aeruginosa* infection appeared to be rebalanced at T1 and T2. Values are median, and p-values were determined using the Wilcoxon signed rank test. ***p < 0.001.

Tregs were impaired in PwCF with versus without chronic pulmonary *P. aeruginosa* infection (7.9% vs. 8.7% of T helper cells), but the between-group difference did not reach statistical significance (p=0.10; **Figure 3**). The increase in Tregs during treatment with ELX/TEZ/IVA was 23.3% in PwCF with chronic *P. aeruginosa* infection and 18.8% in those with no or intermittent *P. aeruginosa* infection. Results from sputum cultures were studied in 37 PwCF with chronic pulmonary *P. aeruginosa* infection over 12 months’ follow-up to determine post-treatment infection status. Data from 36/37 PwCF who provided at least two sequential sputum or throat swab samples were included in this subanalysis (**Table 2**). Sputum samples were available from 26/36 study participants (average 5.3 samples/participant). Throat swabs in case of reduced sputum production after treatment initiation were available from another 10/36 participants (average 3.7 samples/participant). Tregs increased by 39.8% in six PwCF who potentially cleared the infection compared with 16.0% in twenty-one PwCF who had persisting evidence of chronic *P. aeruginosa* infection (**Table 2**). The Treg percentage increased by 32.1% in the nine PwCF who had intermittent post-treatment evidence of *P. aeruginosa* infection.

### 3.5 Effects of ELX/TEZ/IVA on cytokine levels

Twelve T cell-associated cytokines were measured in plasma from 52 participating PwCF (67.5%) before and three months after the initiation of ELX/TEZ/IVA. IL-6 was elevated in 43/52 (82.7%) PwCF at baseline. Plasma IL-6 levels were significantly decreased from baseline at 3-month follow-up (T1) in 38/43 (88.4%) PwCF (median 13.9 pg/mL vs. 3.48 pg/mL; -50.2%; p<0.001), independent of *P. aeruginosa* infection status (p=0.970). ELX/TEZ/IVA had no significant effects on plasma levels of Th1- (IL-2, interferon-γ, TNF-α), Th2- (IL-4, IL-5, IL-13) or Th17- (IL-17A, IL-17F, IL-22) associated cytokines (**Table S1**, **Figure S4**). Although the proportion of Tregs significantly increased during ELX/TEZ/IVA therapy, no statistically significant effects were observed on plasma IL-10 levels (median 2.97 pg/mL at T0 vs. 3.52 pg/mL at T1, p=0.579).

### 3.6 Correlation analysis

During treatment with ELX/TEZ/IVA there were significant improvements in ppFEV_1_, six-minute walk test and sweat chloride, an increase of Tregs, and a reduction in IL-6 levels. Testing the relation between the percentage change of these parameters revealed only weak correlations. After 3 months’ treatment with ELX/TEZ/IVA, increased ppFEV_1_ was not significantly correlated with Treg enhancement (R=0.007, p=0.096), IL-6 decrease (R-0.116, p=0.465) or reduced sweat chloride (R=–0.082, p=0.508). Likewise, changes in sweat chloride levels were not correlated with Treg enhancement (R=0.022, p=0.856) or with reduction of IL-6 levels (R=0.072, r=0.667). There was a weak, but statistically significant, positive correlation between percentage changes in six-minute walk test and Tregs (R=0.304, r=0.024; **Table S2**). Treg enhancement was linked to a downregulation of pro-inflammatory cytokines, predominantly to a reduction of the Th2-related cytokines IL-4 (r=-0.487, p=0.001) and IL-5 (r=-0.415, p=0.005, **Figure S5**).

## 4 Discussion

The results of this longitudinal study show the systemic anti-inflammatory effects of ELX/TEZ/IVA therapy in PwCF, based on an increased proportion of Tregs in peripheral blood and a decreased in plasma IL-6 levels. Furthermore, *P. aeruginosa* infection-mediated Treg impairment appeared to resolve in those with reduced or cleared *P. aeruginosa* infection after treatment with ELX/TEZ/IVA.

The clinical effects of ELX/TEZ/IVA in the current study (a 13 points increase in ppFEV_1_ and a 47% reduction in sweat chloride levels) were consistent with results from the phase 3 trial (1). Moreover, treatment with ELX/TEZ/IVA was associated with a marked increase in Treg percentages (+19%) and, to a lesser extent, a decrease in effector T helper cells (–6%). These changes remained stable at 6-month follow-up. In this study, Tregs were defined as CD4^+^CD127^low^CD25^+^cells and showed a high expression of FoxP3 in a subcohort of 10 PwCF. Treg enhancement was observed in PwCF independently of CRP/leukocyte levels suggesting that Treg enhancement is not only based on a simple shift from effector T helper cells to Tregs. Additional mechanisms might favor an induction of Tregs. Previous studies reported a general Treg impairment in blood and bronchoalveolar lavage fluid from children with CF compared with healthy controls (10, 11). Reduced Tregs have also been found in spleens and lungs from CFTR^-/-^ mice compared with CFTR^+/+^ littermates (10, 23). In addition, pharmacologic inhibition of CFTR dampened Tregs in cultures of human PBMCs indicating a direct link between Treg quantities and CFTR function (10). Therefore, restoration of CFTR function might trigger the Treg enhancement seen during treatment with ELX/TEZ/IVA. We previously did not find any differences in lymphocyte subsets, including Tregs, between PwCF receiving versus not receiving mono or dual combination CFTR modulator therapy (18). However, that cross-sectional study was potentially insufficient to detect differences in immune cell subsets due to missing samples for a longitudinal analysis. Moreover, treatment with mono or dual combination CFTR modulators are less effective to improve CFTR function compared with the triple-combination ELX/TEZ/IVA (18, 19). Experimental data by Gu et al. demonstrated that the subset of CD39^+^ Tregs maintained its suppressive Treg function and FoxP3 expression under inflammatory conditions while CD39^-^Tregs lost their FoxP3 expression (22). We observed a higher proportion of CD39^+^Tregs (in % of Tregs) in response to ELX/TEZ/IVA therapy which probably represents a favorable outcome. CD39^+^Tregs exhibited an enhanced FoxP3 expression compared with CD39^-^Tregs at baseline. At 3-month follow-up, FoxP3 expression levels of CD39^+^/CD39^-^Tregs remained stable. Thus, we detected no disproportional increase of FoxP3 expression under the reduced inflammatory environment that we observed in pwCF receiving ELX/TEZ/IVA therapy. Perhaps, we were unable to find significant differences due to a low number of available samples for FoxP3 staining (n=10) or other, unknown factors regulate FoxP3 expression in our real-world cohort.

Changes downstream of CFTR dysfunction might also play a role in Treg homeostasis. CFTR dysfunction has been associated with an imbalance of sphingolipids (24, 25). Sphingolipids such as ceramides are not only structural components of cell membranes, but also bioactive molecules that are, for example, involved in T cell differentiation (26–28). Interestingly, Treg frequencies were increased in acid sphingomyelinase-deficient mice compared with wildtype mice, and in human PBMC after *in vitro* treatment with acid sphingomyelinase inhibitors such as sertraline (26, 27). Quantification of ceramide subtypes showed a reduced ratio of ceramide species C16/C24 in these cells after treatment (supplementary material of 26). In the context of CF, an elevated ratio of ceramide subtypes C16/C24 correlates with inflammation and disease severity (24, 29). Recently, we reported a 35.5% decrease in the C16/C24 ratio in blood plasma from PwCF treated with ELX/TEZ/IVA (30). These sphingolipid-modulating effects of ELX/TEZ/IVA therapy could potentially be involved in the induction of Tregs in PwCF in the present study.

Tregs have been found to be impaired in blood and bronchoalveolar lavage from PwCF who have chronic *P. aeruginosa* infection and in a mouse model of chronic *P. aeruginosa* lung infection (10, 18, 31). *P. aeruginosa* seems to regulate Treg differentiation independent of direct cell-cell interactions. In particular, cell-free supernatants from virulent and flagellin-deficient *P. aeruginosa* strains impaired Tregs *in vitro* (10). In the present study, Tregs were impaired in PwCF who had chronic *P. aeruginosa* infection at baseline, but differences compared to PwCF who did not have chronic *P. aeruginosa* infection were not statistically significant. So far, conflicting data exist concerning the effects of CFTR modulators on CF-related lung infections. An immediate reduction in *P. aeruginosa* density followed by a rebound at pre-treatment levels has been observed in PwCF treated with ivacaftor (14, 32). Recent studies reported a significant decrease of culture positivity for *P. aeruginosa* in PwCF treated with ELX/TEZ/IVA for 6-12 months (33, 34). We observed a culture clearance of *P. aeruginosa* in 17% of participants in the current study, and intermittent evidence of *P. aeruginosa* infection in another 25% of PwCF with previous chronic *P. aeruginosa* infection. In the present study, PwCF who experienced reduced or cleared chronic *P. aeruginosa* infection were those who showed the strongest increases in Treg percentages, emphasizing the important role of *P. aeruginosa* infection in regulating Treg homeostasis.

Our analysis of T cell-associated systemic cytokines showed a marked reduction in plasma IL-6 levels in PwCF with and without chronic *P. aeruginosa* infection, but stable levels of several other pro-inflammatory cytokines. Thus, we could not attribute anti-inflammatory effects to a reduction of a specific Th2-, Th1- or Th17-type of inflammation in this study. Elevated levels of IL-6 have been detected in both plasma and in bronchoalveolar lavage fluid, and represent a characteristic component of the pro-inflammatory environment in CF that seems to be significantly dampened by ELX/TEZ/IVA (6, 7, 35). Induction of IL-10 has been reported from human PBMCs treated with dual combination CFTR modulators *in vitro* (15). However, in our study, increases of anti-inflammatory plasma IL-10 did not reach statistical significance. Another group reported reduced levels of pro-inflammatory IL-6, IL-8 and IL-17A and stable levels of six other cytokines in blood from PwCF after ELX/TEZ/IVA therapy (33). Lepissier et al. found reduced IL-8 and IL-1β sputum levels in adolescents with mild CF lung disease under ELX/TEZ/IVA therapy (36). These data suggest that CFTR modulators dampen pro-inflammatory cytokines that are predominately associated with neutrophilic inflammation.

In almost all participating PwCF, treatment with ELX/TEZ/IVA improved ppFEV_1_, sweat chloride levels and six-minute walk test. However, correlations between these outcome parameters were limited and not statistically significant, indicating a high variability in treatment responses to ELX/TEZ/IVA. Lung function and (CD39^+^) Tregs were positively correlated at baseline and in previous studies (10, 18), but there was no relationship between the percentage change in ppFEV_1_ and Tregs in response to ELX/TEZ/IVA therapy in the present study. There was a positive correlation between the percentage change in six-minute walk test and Tregs at 3 months, suggesting that Treg enhancement may contribute to the clinical improvement in response to ELX/TEZ/IVA. Weak correlations between outcome parameters have been reported by other groups analyzing various clinical parameters in PwCF receiving ELX/TEZ/IVA (37, 38). Possible explanations are a heterogenous study population in terms of pre-existing structural lung damage, infection status and established therapy with mono or dual combination CFTR modulators, different time intervals until full therapeutic effects are achieved and/or complementary, non-correlating effects being captured by a parameter.

The current study is the largest to date investigating the composition of immune cell subsets and systemic cytokines in PwCF receiving highly effective triple combination CFTR modulators. However, the study also has some limitations. Although our cohort included PwCF with mild to severe lung disease, the average ppFEV_1_ was lower than in the approval study for ELX/TEZ/IVA, and therefore our results might not be directly applicable to PwCF who have milder disease. Our cohort was heterogeneous regarding several baseline characteristics (ppFEV_1_, genotype, infection status, previous CFTR modulator therapy). Nonetheless, our main finding of Treg enhancement was found in all subgroups. Leukocytosis and elevated CRP were present in several PwCF at baseline due to chronic infection and/or CF-related inflammation. Therefore, studying the natural course and immunological reaction to ELX/TEZ/IVA therapy is complicated by the individual, complex inflammatory state of each study participant. A more detailed characterization of Tregs was outside the initial scope of the present study. The number of stored PBMC samples limited additional retrospective FACS analyses. Furthermore, we did not conduct *in vitro* Treg suppression assays to analyze a possible, altered Treg function in response to ELX/TEZ/IVA therapy. Future studies may provide a more comprehensive analysis of Treg phenotypes and function in PwCF under ELX/TEZ/IVA therapy.

## 5 Conclusions

We report stable Treg enhancement in peripheral blood from PwCF receiving ELX/TEZ/IVA therapy. This effect is probably mediated by a restoration of CFTR function and a reduction in *P. aeruginosa* airway colonization. Our results underline the anti-inflammatory effects of ELX/TEZ/IVA therapy, with implications for future clinical trials evaluating anti-inflammatory therapies for PwCF, especially for those with persistent pulmonary infections.

## Data availability statement

The raw data supporting the conclusions of this article will be made available by the authors, without undue reservation.

## Ethics statement

The studies involving human participants were reviewed and approved by Ethik-Kommission, Universität Duisburg-Essen (no. 17-7365-BO). The patients/participants provided their written informed consent to participate in this study.

## Author contributions

Study conception: SR, CT, DW. Data acquisition and analysis: DW, HU, JR, MS, JP, SS, SSu, MW. Data interpretation: DW, SR, JR, CT. Writing the original manuscript: DW, SR. Revising the work for important intellectual content: SR, CT, DW, SSu, MW, HU, JR, MS. All authors contributed to the article and approved the submitted version.
