# Supplementary material for: Regulatory T cell enhancement in adults with cystic fibrosis receiving Elexacaftor/Tezacaftor/Ivacaftor therapy
Source: Front Immunol. 2023 Feb 16;14:1107437. doi: 10.3389/fimmu.2023.1107437 (PMC9978140; doi:10.3389/fimmu.2023.1107437)
Supplement: Supplementary file 1 [file DataSheet_1.docx]

Supplementary Material

Regulatory T Cell Enhancement in Adults with Cystic Fibrosis Receiving Elexacaftor/Tezacaftor/Ivacaftor Therapy

Dirk Westhölter^1*^, Jonas Raspe^1^, Hendrik Uebner^1^, Johannes Pipping^1^, Mona Schmitz^1^, Svenja Straßburg², Sivagurunathan Sutharsan², Matthias Welsner², Christian Taube^1^, Sebastian Reuter^1^

^1^Department of Pulmonary Medicine, University Hospital Essen- Ruhrlandklinik, Essen, Germany

^2^ Adult Cystic Fibrosis Center, Department of Pulmonary Medicine, University Hospital Essen- Ruhrlandklinik, Essen, Germany

***Correspondence:**Dirk Westhölter, M.D.
[dirk.westhoelter@uk-essen.de](mailto:dirk.westhoelter@uk-essen.de)

# Supplementary Tables

**Table S1.** Systemic cytokines before (T0) and after 3 months of elexacaftor/tezacaftor/ivacaftor therapy (T1; samples were available from 52 participating PwCF). Plasma IL-6 levels were significantly decreased from baseline at 3-month follow-up.

| **Cytokines** | | **T0** | **T1** | **p-value** |
| --- | --- | --- | --- | --- |
| IL-5, pg/mL | | 7.03 [3.37, 17.42] | 11.82 [3.16, 18.33] | 0.868 |
| IL-13, pg/mL | | 4.21 [0, 9.35] | 3.48 [0, 6.70] | 0.185 |
| IL-2, pg/mL | | 1.11 [0, 3.56] | 1.66 [0, 3.14] | 0.819 |
| IL-6, pg/mL | | 13.92 [3.63, 33.46] | 3.48 [0, 9.05] | **<0.001** |
| IL-9, pg/mL | | 0.97 [0, 4.61] | 1.76 [0, 4.04] | 0.928 |
| IL-10, pg/mL | | 2.97 [1.64, 5.54] | 3.52 [1.70, 5.45] | 0.579 |
| IFN-γ, pg/mL | | 14.37 [3.78, 36.74] | 17.54 [5.02, 40.33] | 0.335 |
| TNF-α, pg/mL | | 22.54 [4.55, 61.96] | 25.12 [5.75, 75.49] | 0.556 |
| IL-17A, pg/mL | | 1.75 [0.65, 2.94] | 1.93 [0.60, 3.09] | 0.819 |
| IL-17F, pg/mL | | 2.44 [0, 10.95] | 5.15 [0, 10.88] | 0.450 |
| IL-4, pg/mL | 19.45 [1.69, 54.23] | | 16.72 [2.19, 47.14] | 0.835 |
| IL-22, pg/mL | 4.23 [0.00, 7.58] | | 4.59 [1.93, 8.64] | 0.713 |

Values are median [quartile 1, quartile 3]. p-value determined using Wilcoxin signed rank test. IFN, interferon; IL, interleukin; TNF, tumor necrosis factor.

**Table S2.** Correlation analysis. Spearman correlations of percent predicted FEV_1_ (ppFEV_1_, %), sweat chloride levels (mmol/L), six-minute walk test (m), regulatory T cells (Tregs; CD4^+^, CD25^+^, CD127^-^, as a proportion of T helper cells) and Tregs with CD39^+^ expression (as a proportion of Treg) at baseline (T0, n=77), and for the percentage change in these parameters between T0 and T1. At baseline there were significant correlations between ppFEV_1_ and CD39^+^ Tregs. In addition, there was a significant association between the increase in Tregs between T0 and T1 and the increase in six-minute walk test duration over the same period.

| **T0 Correlation** | **R** | **p-value** |
| --- | --- | --- |
| **T0 Correlation** |  |  |
| ppFEV_1_ & Treg | –0.160 | 0.892 |
| ppFEV_1_ & CD39 Treg | **0.253** | **0.026** |
| ppFEV_1_ & sweat chloride | –0.017 | 0.886 |
| ppFEV_1_ & six-minute walk test | **0.440** | **<0.001** |
| Sweat chloride & Treg | –0.139 | 0.241 |
| Sweat chloride & CD39 Treg | –0.094 | 0.427 |
| Sweat chloride & six-minute walk test | –0.149 | 0.244 |
| Six-minute walk test & Treg | 0.026 | 0.837 |
| Six-minute walk test & CD39 Treg | 0.140 | 0.265 |
| **ΔT0-T1 Correlation** |  |  |
| Δ ppFEV_1_ & Δ Treg | 0.007 | 0.956 |
| Δ ppFEV_1_ & Δ CD39 Treg | –0.004 | 0.971 |
| Δ ppFEV_1_ & Δ sweat chloride | –0.082 | 0.508 |
| Δ ppFEV_1_ & Δ six-minute walk test | 0.003 | 0.983 |
| Δ Sweat chloride & Δ Treg | 0.022 | 0.856 |
| Δ Sweat chloride & Δ CD39 Treg | –0.050 | 0.682 |
| Δ Sweat chloride & six-minute walk test | 0.148 | 0.300 |
| Δ Six-minute walk test & Δ Treg | **0.304** | **0.024** |
| Δ Six-minute walk test & Δ CD39 Treg | 0.205 | 0.134 |

Δ, delta (change); ppFEV_1_, percent predicted forced expiratory volume in 1 second; Treg, regulatory T cell.

# Supplementary Figures


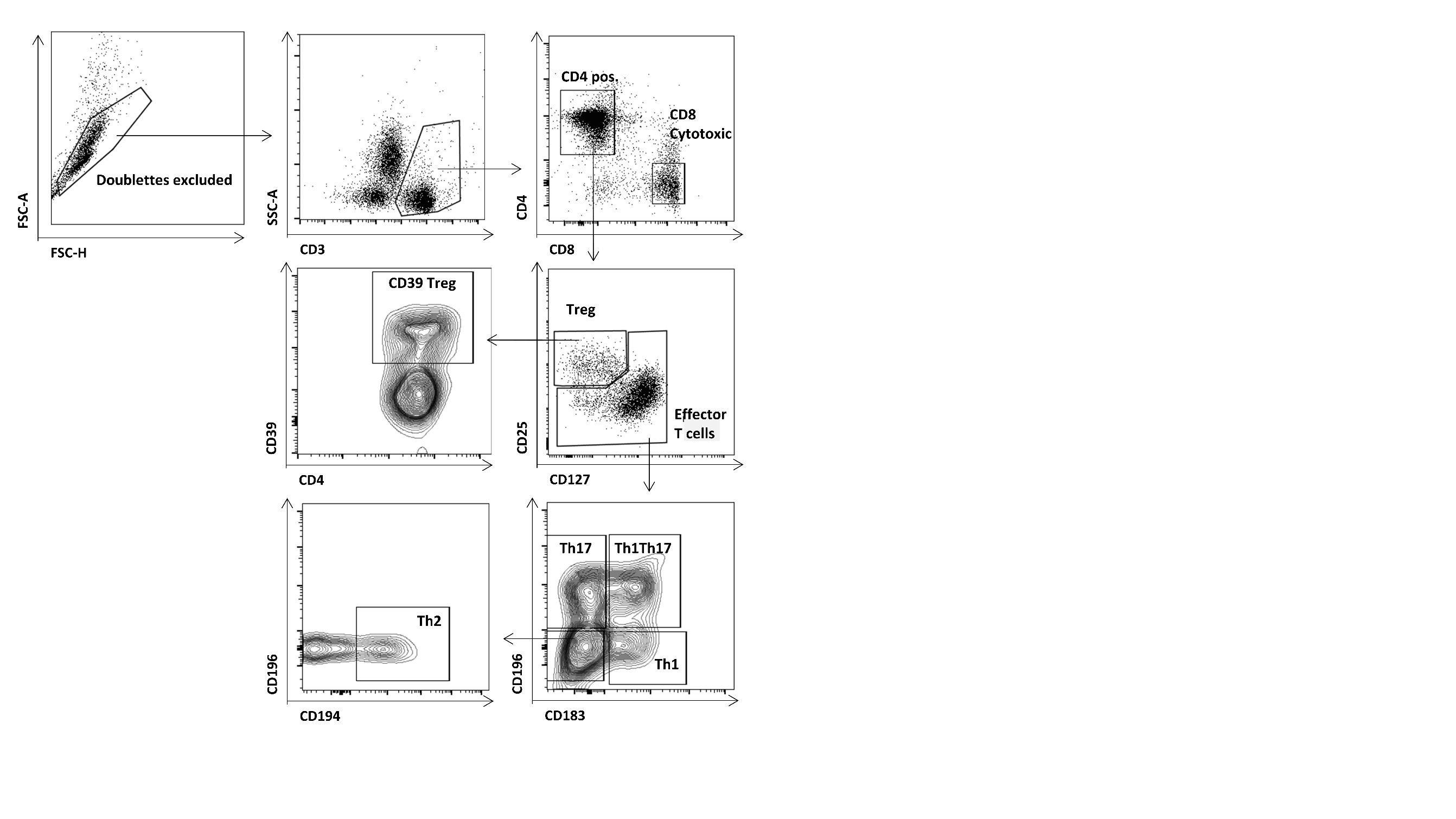


**Figure S1.** Gating strategy to determine lymphocyte subsets (adapted from Rühle et al. (18, 20)). First depris and doublettes were excluded based on size in the forward scatter area (FSC-A) versus height (FSC-H). T lymphocytes were identified as CD3^+^ and side ward scatter (SSC) low cells. Within T lymphocytes, T helper cells were characterized as CD4^+^ cells and cytotoxic T cells as CD8^+^ cells. T helper cells were further characterised as CD127^low^CD25^+^ regulatory T cells (Tregs) and effector T cells. Regulatory T cells (Tregs) were subdivided into CD39^+^ “stable Tregs” and CD39 low conventional Tregs (22). T helper effector cells were further characterized based on the expression of CD183, CD194 and CD196 in Th1 (CD183^+^/CD196^-^), Th1/Th17 (CD183^+^/CD196^+^), Th17 (CD183^-^/CD196^+^) and Th2 cells (CD183^-^/CD196^-^/CD194^+^).


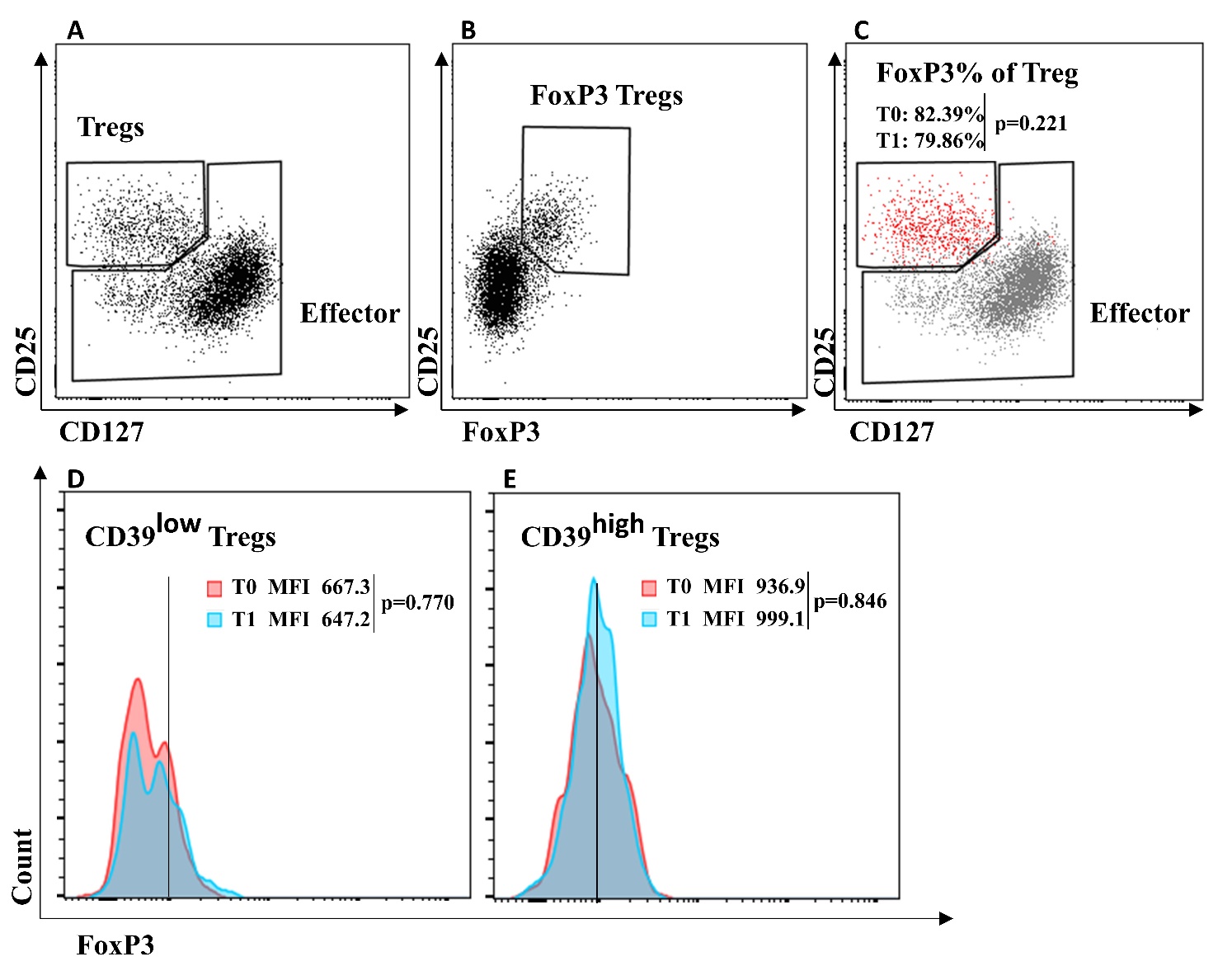


**Figure S2.** Treg characterisation. FoxP3 expression of CD127^low^CD25^+^ regulatory T cells (Tregs) in a subcohort of pwCF (n=10, paired data). A: Tregs were characterised as CD127^low^CD25^+^ cells among CD4^+^ T helper cells in this study. B: Intracellular staining against FoxP3 and gating of CD4^+^CD25^+^FoxP3^+^ cells. C: Backgating of CD4^+^CD25^+^FoxP3^+^ cells (red) to confirm FoxP3 expression in CD127^low^CD25^+^ Tregs. FoxP3 positivity was found in about 80% of CD127^low^CD25^+^ Tregs and remained stable before (T0) and after (T1) ELX/TEZ/IVA therapy. D: CD39 characterizes a stable subset of Tregs. Mean fluorescence intensity (MFI) of FoxP3 measured in CD39^low^Tregs was stable before (T0) and after (T1) starting on ELX/TEZ/IVA. E: MFI (FoxP3) remained stable before and after ELX/TEZ/IVA therapy in CD39^high^Tregs. MFI (FoxP3) was overall higher in CD39^high^Tregs compared with CD39^low^Tregs. Statistics: Values are mean. Wilcoxon signed-rank test.


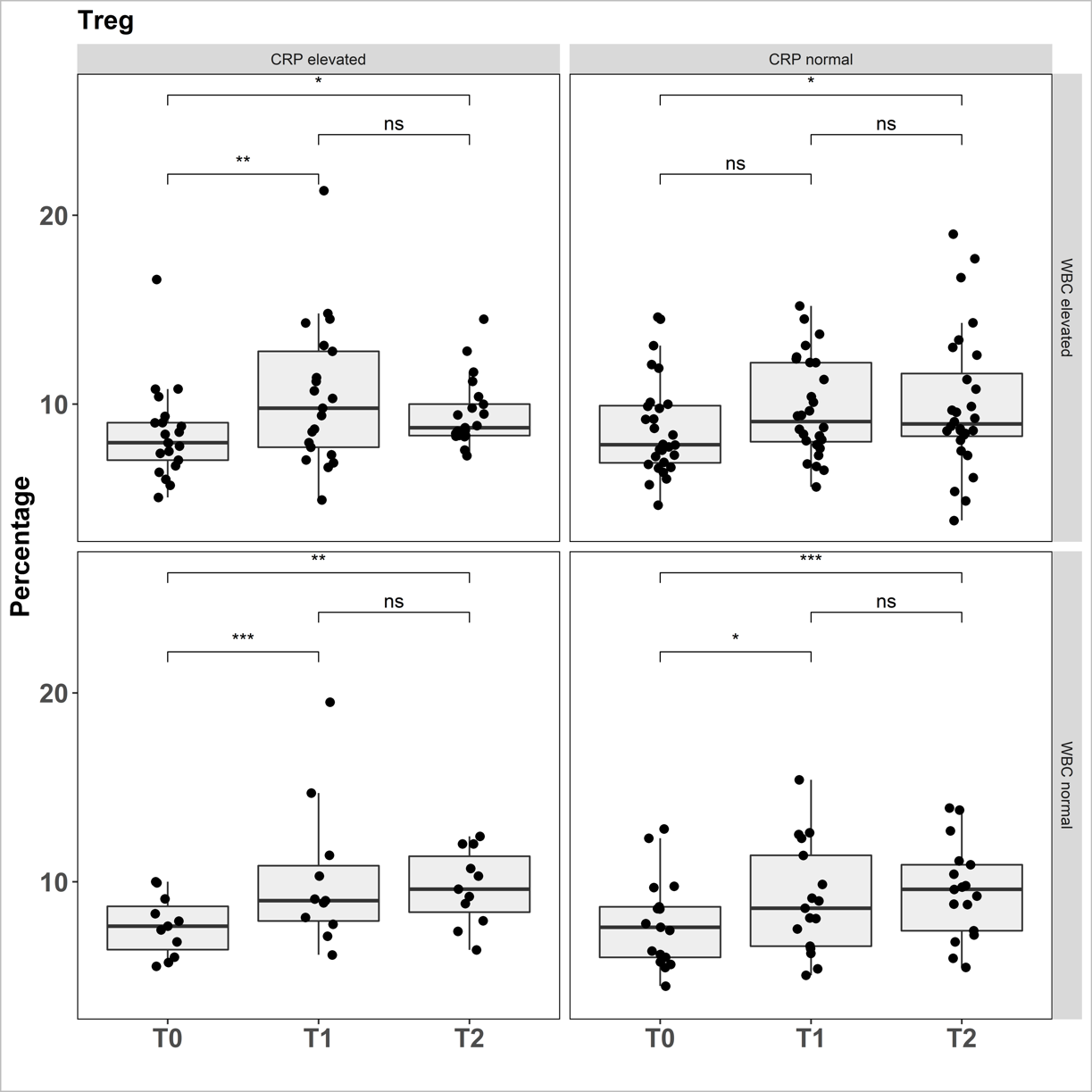


**Figure S3.** Stratification of CD25^+^CD127^-^ regulatory T cells (Tregs; as a proportion of T helper cells [T_h_]) by C-reactive protein (CRP) and leukocytes/white blood cell count (WBC). Treg enhancement was observed in all subgroups, with or without elevated CRP and/or leukocytosis. Statistics: median values, Wilcoxon signed-rank test. ns, not statistically significant. *p<0.05; **p<0.01; ***p<0.001.


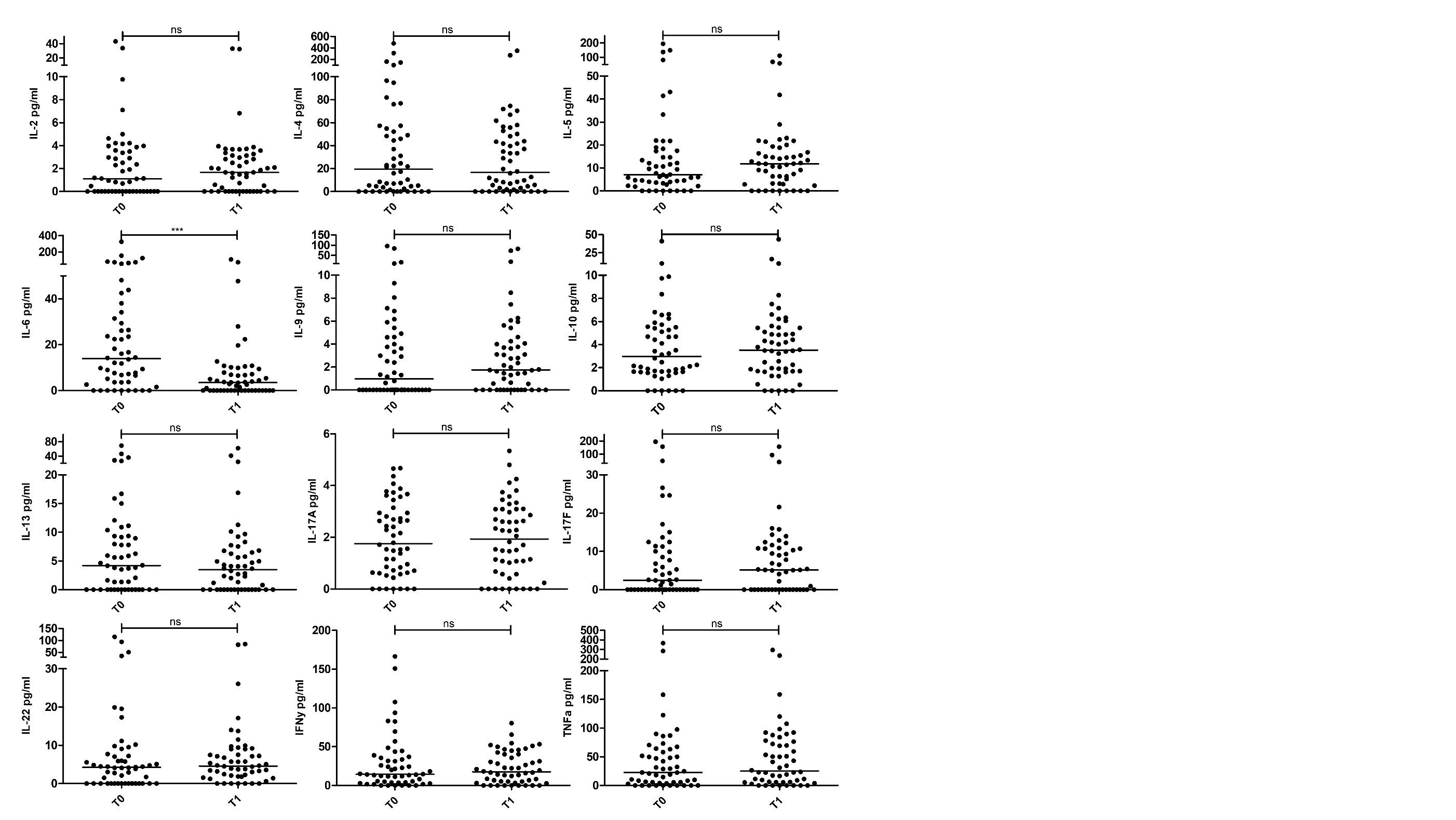


**Figure S4.** Cytokine measurements in plasma from 52 PwCF before (T0) and 3 months after (T1) initiation of elexacaftor/tezacaftor/ivacaftor therapy. Interleukin [IL]-6 levels were significantly reduced at T1. Statistics: median values, Wilcoxon signed rank test. ns, not statistically significant.


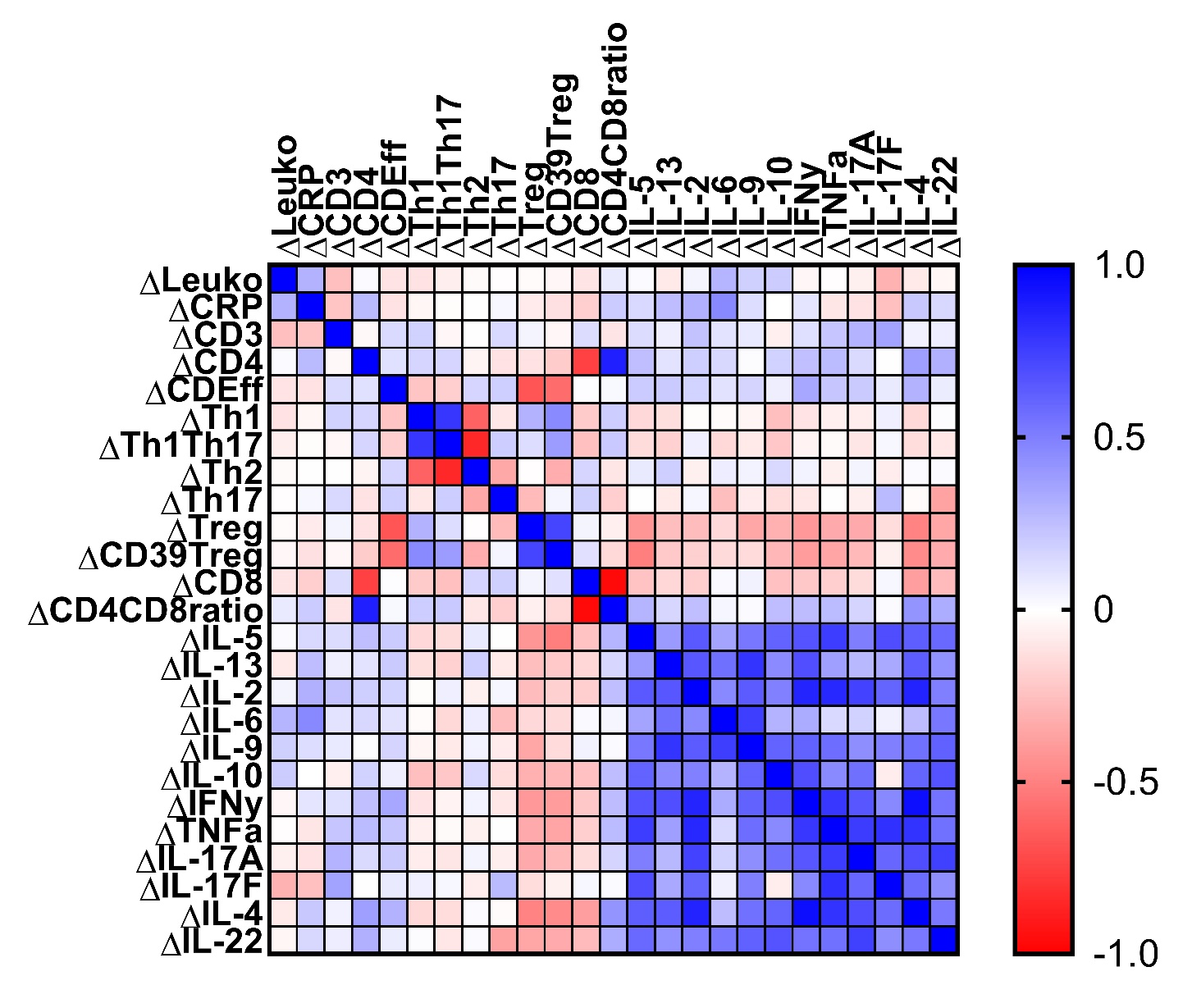


**Figure S5**. Correlation matrix of delta change of systemic cytokines and lymphocyte subsets between T0 and T1 (before and 3 months after receiving ELX/TEZ/IVA). Colour intensity is proportional to the correlation coefficients (blue r=1; red r=–1). Overall, Δcytokines were correlated with each other. ΔCRP and ΔIL-6 showed significant correlation (r=0.460, p=0.036). Increase of Treg/ CD39Tregs was associated with a downregulation of cytokines, predominately Th2-associated cytokines (e.g. ΔCD39Treg&ΔIL-5, r=-0.506, p<0.001; ΔTreg&ΔIL-4, r=-0.487, p=0.001). Statistics: Spearman correlation.
